# Effects of fragility fracture integrated rehabilitation management on mobility, activity of daily living and cognitive functioning in elderly with hip fracture

**DOI:** 10.12669/pjms.36.5.2412

**Published:** 2020

**Authors:** Anam Aftab, Waqar Ahmed Awan, Shaista Habibullah, Jae Young Lim

**Affiliations:** 1 Dr. Anam Aftab, PhD., Riphah College of rehabilitation Sciences, Riphah International University, Islamabad, Pakistan; 2 Dr. Waqar Ahmad Awan, PhD., Riphah College of rehabilitation Sciences, Riphah International University, Islamabad, Pakistan; 3 Dr. Shaista Habibullah, PhD. National Institute of Rehabilitation Medicine, Islamabad Pakistan; 4 Dr. Jae Young Lim, PhD. Seoul National University, Bundang Hospital, South Korea

**Keywords:** Activities of Daily Living, Cognition, Hip Fractures, Mobility Limitation, Occupational Therapy, Physical Therapy

## Abstract

**Objective:**

To determine the effectiveness of Fragility Fracture Integrated Rehabilitation Management (FIRM) on mobility, activity of daily living and cognitive functioning in elderly with hip fracture.

**Methods:**

A randomized control trial was conducted at Seoul National University Bundang Hospital, South Korea from August 2017 to January 2018. Patients of both genders with the age 65-95 years, diagnosed cases of hip fracture specifically fractures neck of femur, intertrochanteric, subtrochantric, patients who got bipolar hemiarthroplasty, total hip replacement arthroplasty, reduction and internal fixation were included in this study. A total of n=39 sample was collected through non probability convenience sampling technique and randomly divided into Fragility Integrated Rehabilitation Management (FIRM) group (n=20) and Conventional Physical therapy (CPT) group (n=19). The data was collected through KOVAL for walking ability, modified barthal index (MBI) for behaviors related to activities of daily living (ADLS) and mini mental status examination (MMSE) for cognitive functions at baseline on 2^nd^ postoperative day and after 10^th^ FIRM session on 15^th^ postoperative day.

**Results:**

The mean age of study participants was 82.07±6.00 years. The post intervention comparison did not show any significant difference (*p*>0.05) in walking ability, overall ADLs and cognitive functioning. But FIRM group showed significant improvement in stair climbing {0(5) ver. 2(7.5), *p*=0.049} and ambulation or walker use {8(5) ver. 2(4), *p*=0.037}, as compared to CPT group.

**Conclusion:**

Both groups improved in indoor mobility with walker and crutches as well as activities of daily living. But FIRM showed more improving ambulation with walker and stair climbing. While cognitive functioning was observed only in FIRM group.

## INTRODUCTION

Fragility fractures; low-impact injuries that results from a fall from standing or lesser height, which shows a serious public health issue.^1^ Although the quality of the surgical and perioperative treatment of hip fracture has improved, but physical and functional recovery after surgery and acute care remains deficient. Before hip fracture, 11% of community-dwelling elderly individuals are bed-ridden and 16% are in long-term-care facilities.^2^,^3^ Within one year after sustaining a hip fracture, individuals experience a very serious decrease in the quality of their life, and the mortality rate in this group is as high as 36%.^4^

With medical advancements the surgery quality and preoperative management protocol for hip fracture patients have improved a lot but there is still a lot of space for improvement in post surgical care protocols.^3^ One year or more after the hip surgery the patients experience a significant decline in the quality of life of patients which in turn increase the mortality rate.^4^ Complete rehabilitation for hip fracture consists of physical therapy (PT), occupational therapy (OT), fall prevention, nutritional modifications, psychiatric support, complication prevention, and discharge scheduling with environmental adjustments.^2^,^5^

The Korean Fragility Fracture Rehabilitation Study Group has since developed a fragility fracture integrated rehabilitation management (FIRM) plan for patients with hip fracture. Fragility Integrated Rehabilitation, coordinated with social care is a crucial piece of the care pathway to patients who have endured a fragility fracture and can essentially decrease expenses and guarantee better outcome for the elderly patients who endure hip fractures.^2^,^6^

FIRM being a new advanced technique of rehabilitation which can be considered an advanced version of geriatric inter disciplinary rehabilitation with lot of systematic additions to that. However, no comparative study on effectiveness of FIRM compared to conventional rehabilitation was available in literature in which rehabilitation was performed by interdisciplinary teams. This study was conducted to determine the effectiveness of Fragility Fracture Integrated Rehabilitation Method (FIRM) on mobility, activity of daily living and cognitive functioning as compared to conventional physical therapy in elderly with hip fracture.

## METHODS

**Fig.1:**
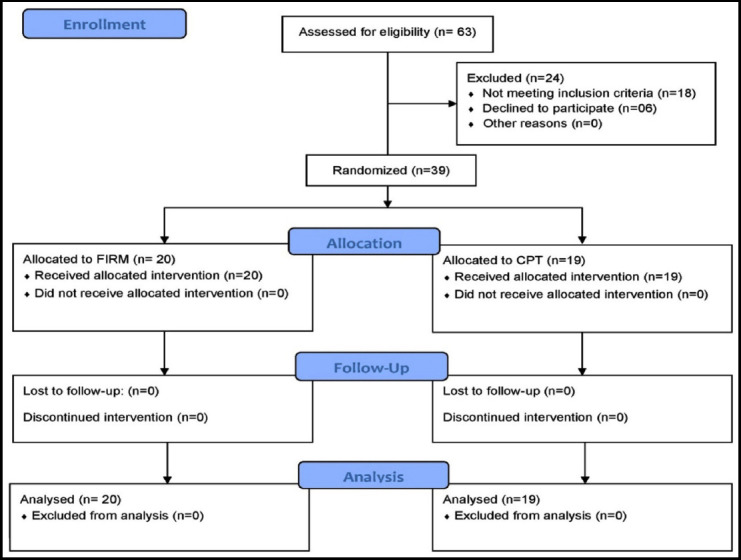
CONSORT diagram.

A randomized control trial (Clinical Trial # NCT03430193) was conducted at Seoul National University Bundang Hospital South Korea from August 2017 to January 2018. The study was conducted after the ethical approval (IRB No. B-1603/337-002, dated May 15, 2015). Patients of both genders with the age 65-95 years, diagnosed cases of hip fracture specifically fractures neck of femur, intertrochanteric, subtrochantric, patients who got bipolar hemiarthroplasty, total hip replacement arthroplasty, reduction and internal fixation were included in this study. Patients who underwent surgery for more than once for a hip fracture, femoral shaft fracture, acetabular and peri-prosthetic fracture, isolated fracture of greater or lesser tuberosity, multiple fractures and revision operation were excluded in this study. A total of n=39 sample was collected through non probability convenient sampling technique and randomly divided into by Fragility Integrated Rehabilitation Management (FIRM) group (n=20) and Conventional Physical therapy (CPT) group (n=19) (Fig.1).

FIRM group received comprehensive rehabilitation program provided by rehabilitation physician, physical therapist, occupational therapist, nutritionist, clinical nurse and social worker. Physical therapy and occupational therapy aimed to improve mobility and activities of daily living. Each participant was admitted for 15 days after surgery and received 10 physical therapy (PT) sessions (FIRM # 1-10) and 4 occupational therapy (OT) sessions (FIRM #4, 6, 8, 10). PT sessions included weight-bearing, strengthening, gait training, aerobics, and functional exercises. The duration of each session was at least 40 Minutes. Occupational therapy included training of activities of daily living (ADLs) transfer, sit to stand, bed mobility, dressing, self-care retraining, and use of adaptive equipment. Multidisciplinary rehabilitation members also provided comprehensive patients education.^2^ Conventional postoperative rehabilitation involved PT for 40 min/day, ward education, fall prevention, discharge planning, including in-hospital, postoperative usual orthopedic care. Ward education included techniques about carrying out clothing, carrying out transfer, education about standing exercises, bed exercises and strengthening exercises with elastic band and toileting. The discharge notes included explanation about posture, education to prevent falls and home environment modifications.

Data was collected at baseline on 2^nd^ postoperative day and after 10^th^ FIRM session on 15^th^ postoperative day after intervention. The written informed consent was taken from all participants, was according to Declaration of Helsinki. The demographic data at the baseline was obtained in term of age, gender and BMI. The base line and after intervention, data was collected through KOVAL for walking ability, modified barthal index (MBI) for behaviors related to activities of daily living (ADLS) and mini mental status examination (MMSE) for cognitive functions. The sphiro-wilk test showed that data was normally distributed without having significant outlier observed on box plots. So parametric tests were used, including paired samples t-test for within group analysis and independent samples t-test for between groups comparison were used respectively. Non-parametric tests were used for skewed data including Wilcoxon Sign Rank test and Mann Whitney U Test for within and between group analyses respectively. The data was analyzed on SPSS ver. 21 and level of significance was set at 95% CI (*p≤0.05*).

## RESULTS

**Fig.2:**
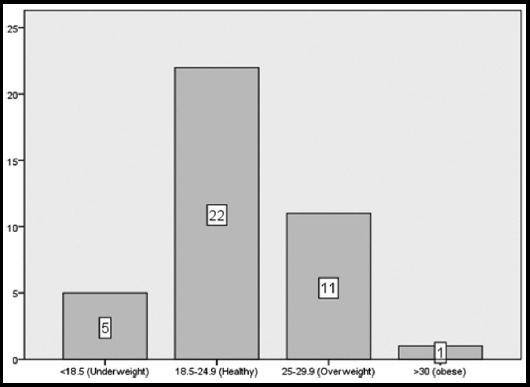
BMI categories of study participants.

A total of n=7 male and n=32 female participants having mean age of 82.07±6.00 years were the participants. The average BMI (23.15±3.93) showed mostly individual were healthy range (Fig.2).

The results of pre and post analysis showed that both group significantly improved walking ablity measured on KOVAL (*p*<0.05). The activties of daily living (ADLs) measured on MBI showed that bathing, toileting, stair climibing, dressing, ambulation and chair & bed transfer showed significant improvement in both groups (*p*<0.05). The overall score of ADLs also improved significatly in both groups (*p*<0.05) after intervention. The cognitive functioning measured in mini mental status examination (MMSE) showed significant improvement only in FIRM group (*p*<0.05) (Table-I).

**Table-I:** Within Group Changes (KOVAL, MBI & MMSE).

Variables	Groups	Pre	Post	Z	p-value

		Mean±SD/ Median(IQR)	Mean±SD/ Median(IQR)		
KOVAL	Conventional	7(0.25)	6(0)	-3.87	<0.001***
	FIRM	6(1)	6(0)	-2.12	0.034
Personal Hygiene	Conventional	4(1.25)	4(1)	-1	0.317
	FIRM	4(1)	4.5(1)	-1.63	0.102
Bathing	Conventional	1(3)	3(4)	-2.72	<0.001***
	FIRM	1(3)	3(2)	-2.85	<0.001***
Feeding	Conventional	10(5)	10(2)	-1.08	0.276
	FIRM	10(0)	10(0)	-1.34	0.189
Toilet	Conventional	2(5.75)	5(6)	-2.53	0.011**
	FIRM	5(8)	8(5.25)	-2.73	<0.001***
Stair Climbing	Conventional	0(0)	0(5)	-2.21	0.027**
	FIRM	0(0)	2(7.5)	-3.09	<0.001***
Dressing	Conventional	2(3)	5(3)	-2.58	0.010**
	FIRM	5(3)	6.5(3)	-3.55	<0.001***
Bowel Control	Conventional	10(5.75)	10(5)	-0.13	0.892
	FIRM	10(5)	10(1.5)	-1.89	0.058
Bladder Control	Conventional	10(8.50)	10(2)	-0.81	0.416
	FIRM	10(2)	10(2)	-1.28	0.197
Ambulation or Walker	Conventional	0(4.25)	8(5)	-3.33	<0.001***
	FIRM	8(8)	12(4)	-3.32	<0.001***
Wheelchair	Conventional	0(0)	0(0)	0	1.00
	FIRM	0(0)	0(0)	0	1.00
Chair and Bed Transfer	Conventional	3(12)	8(4)	-2.82	<0.001***
	FIRM	8(9)	12(4)	-2.81	<0.001***
MBI Total	Conventional	43.52±19.96	58.63±20.69	-	<0.001***
	FIRM	51.45±23.31	69.60±19.98	-	<0.001***
MMSE	Conventional	18.42±7.81	18.94±7.71	-	0.471
	FIRM	20.75±6.61	22.15±5.92	-	0.020**

Level of significance: p<0.001***& p<0.05**.

The post intervention comparison did not show any significant difference (*p*>0.0) in walking ability, overall ADLs and cognitive functioning. But FIRM group showed significant improvement in stair climbing {0(5) ver. 2(7.5), *p*=0.049} and ambulation or walker use {8(5) ver. 2(4), *p*=0.037}, as compare to CPT group (Table-II).

**Table-II:** Between Group comparison (KOVAL, MBI & MMSE).

Variables	Groups	Pre			Post		

		Mean±SD/ Median(IQR)	U-test	p-value	Mean±SD/ Median(IQR)	U-test	p-value
KOVAL	Conventional	7(0.25)	85.50	0.001	6(0)	189.50	0.979
	FIRM	6(1)			6(0)		
Personal Hygiene	Conventional	4(1.25)	152.50	0.252	4(1)	133.50	0.081
	FIRM	4(1)			4.5(1)		
Bathing	Conventional	1(3)	189	0.976	3(4)	186.50	0.918
	FIRM	1(3)			3(2)		
Feeding	Conventional	10(5)	168.50	0.458	10(2)	140.50	0.078
	FIRM	10(0)			10(0)		
Toilet	Conventional	2(5.75)	175	0.663	5(6)	153.50	0.280
	FIRM	5(8)			8(5.25)		
Stair Climbing	Conventional	0(0)	170.50	0.298	0(5)	123.50	0.049**
	FIRM	0(0)			2(7.5)		
Dressing	Conventional	2(3)	158.50	0.328	5(3)	127.50	0.060
	FIRM	5(3)			6.5(3)		
Bowel Control	Conventional	10(5.75)	186	0.899	10(5)	164	0.370
	FIRM	10(5)			10(1.5)		
Bladder Control	Conventional	10(8.50)	187.50	0.945	10(2)	182	0.783
	FIRM	10(2)			10(2)		
Ambulation or Walker	Conventional	0(4.25)	121	0.040	8(5)	120	0.037**
	FIRM	8(8)			12(4)		
Wheelchair	Conventional	0(0)	190	1	0(0)	190	1
	FIRM	0(0)			0(0)		
Chair and Bed Transfer	Conventional	3(12)	147	0.214	8(4)	140.50	0.146
	FIRM	8(9)			12(4)		
MBI Total	Conventional	43.52±19.96	-	0.262	58.63±20.69	-	0.101
	FIRM	51.45±23.31			69.60±19.98		
MMSE	Conventional	18.42±7.81	-	0.321	18.94±7.71	-	0.157
	FIRM	20.75±6.61			22.15±5.92		

Level of significance: p<0.001***& p<0.05**.

## DISCUSSION

The objective of the study was to determine the effectiveness of Fragility Fracture Integrated Rehabilitation Management (FIRM) on mobility, activity of daily living and cognitive functioning as compared to conventional physical therapy (CPT) in elderly with hip fracture. The results suggested that participants in both group showed significant improvement in indoor mobility with walker and crutches and activities of daily living. But cognitive functioning was significantly improved only in FIRM group. While comparing both groups after 10^th^ session FIRM group showed significant improvement in stair climbing and mobility with walker or crutches as compared to conventional PT group.

The results of current study suggested significant improvement in walking with crutches or walker and stair climbing ability, in FIRM group as compared to CPT group. Several studies^2^,^5^,^7^ supported the current study, including a randomized controlled trial performed to evaluate the effectiveness of fragility fracture integrated rehabilitation management (FIRM) following hip fractures in the elderly patients, reported significantly increased mobility and ADL scores, and improved physical functioning in the intervention group as measured by Koval Scale.^2^ Norstrom-P et al. reported that systematic rehabilitation performed by geriatric interdisciplinary teams, improved the physical function and mobility when compared with conventional care.^7^

There are several studies suggesting that comprehensive rehabilitation program significantly improved mobility and lower the risk of depression among hip fracture patients. In a single-center controlled trial on n=1077 geriatric hip fracture patients aging from 70 years or older, complete geriatric rehabilitation for four months improved mobility significantly as compared to usual orthopedic care.^8^ Shyu Yi et al. compared three groups of elderly patients (n=229) with hip fracture treated with different approaches: usual care, interdisciplinary care (geriatric consultation, continuous rehabilitation, and discharge planning), and comprehensive care (interdisciplinary care plus nutrition consultation, depression management, and fall prevention). This research found a lower risk of depression and malnutrition in the comprehensive care group than in the interdisciplinary care group one year after discharge. Therefore, better functional outcomes can be expected following the provision of a comprehensive postoperative rehabilitation program to hip fracture patients.^9^ In current short duration study FIRM protocol that included comprehensive rehabilitation program provided by rehabilitation physician, physical therapist, occupational therapist, nutritionist, clinical nurse and social worker.

In a study by Yea-Ing L. Shyu et al, intervention group elderly hip fracture patients receiving an interdisciplinary program of geriatric consultation, continuous rehabilitation and discharge planning was compared to control group receiving conventional care. The results showed significant improvement in walking ability and fewer depressive symptoms in the intervention group.^10^ In another study participants receiving geriatric interdisciplinary rehabilitation at home with those receiving conventional rehabilitation, showed no significant difference in walking ability and use of walking device, however the time spent in hospital was significantly shorter in the geriatric interdisciplinary home rehabilitation group.^5^

The current study did not show significant difference in FIRM and CPT group regarding improvement of cognitive functioning on MMSE score after 10^th^ session. But a study reported, the results of two year follow up of interdisciplinary rehabilitation program as compared to those receiving routine care, that 75% less likelihood of post-discharge cognitive impairment in patients receiving.^11^ Depression and poor cognitive functioning also cause poor physical functioning and daily life activities in hip fracture patients.^12^

### Limitation of the study

This study observed short term effects in 2-week post-operative hospital stay with small sample size. To see long term effects of 2-week FIRM protocol, the study should be conducted with multiple follow ups with large sample size at different duration for at least six months after discharge following home plan which is a part of FIRM protocol. This was a single centered study conducted in South Korea, a developed country, so results of the study cannot be generalized to developing country where lack of facilities and human resource are the major problems in the delivery of FIRM protocol to patients with hip fracture.

## CONCLUSION

Both groups improved in indoor mobility with walker and crutches as well as activities of daily living. But FIRM showed more improving ambulation with walker and stair climbing. While cognitive functioning was observed only in FIRM group. It is suggested that long term effects of FIRM protocol should be observed on cognition as well as on quality of life. It is also recommended that FIRM protocol must be evaluated in developing countries like Pakistan to promote quality of life of elderly patients with hip fracture.

### Authors’ Contribution

**AA & JYL** conceived, designed and data collection.

**AA & WAA** did manuscript writing, statistical analysis & editing of manuscript.

**WAA** is responsible and accountable for the accuracy or integrity of the work.

**SH** did review and final approval of manuscript.
